# Artificial intelligence as a source of psychotherapy-related psychoeducation: a cross-sectional content analysis of information on eye movement desensitization and reprocessing

**DOI:** 10.3389/fpsyt.2026.1937506

**Published:** 2026-09-09

**Authors:** İbrahim Karakaya, Onur Okan Demirci

**Affiliations:** Private Psychiatric Practice, Istanbul, Türkiye

**Keywords:** artificial intelligence, digital mental health, EMDR therapy, informational quality, large language models, psychoeducation, readability

## Abstract

**Background:**

Due to structural and socioeconomic barriers to accessing the healthcare system, individuals are increasingly turning to online platforms to obtain health-related information. With the advancement of artificial intelligence (AI), the use of AI for this purpose is also on the rise. It can also be said that seeking information from AI regarding psychotherapies is increasingly common.

**Objective:**

This study aimed to assess the informational quality and readability of AI-generated psychoeducational content about psychotherapy. To this end, we evaluated the informational quality and readability of LLM-generated Eye Movement Desensitization and Reprocessing (EMDR) psychoeducational content across different models.

**Methods:**

In this cross-sectional content analysis, EMDR-related search queries were identified through Google Trends, converted into patient-facing questions, and subsequently organized into four thematic categories, with five questions selected from each category for evaluation using ChatGPT (GPT-4o), Gemini (1.5 Pro), and DeepSeek (V3). Each question was entered in three separate sessions, yielding 180 responses. The informational quality was assessed by two blinded psychiatrists using the Ensuring Quality Information for Patients (EQIP) instrument. Readability was evaluated using the Flesch–Kincaid Grade Level (FKGL) and Flesch Reading Ease (FRE) indices. Statistical analyses included linear mixed-effects models and ANOVA.

**Results:**

The overall mean EQIP score was 56.08 (SD = 4.43). Informational quality differed significantly across the models (p < 0.001), and no significant differences were observed across EMDR-related question categories. The highest informational quality scores were obtained by Gemini, followed by DeepSeek and ChatGPT. DeepSeek generated the most readable responses. Higher EQIP scores were seen to be associated with greater readability and longer response length. All the evaluated models produced responses that exceeded the commonly recommended readability levels for patient education materials. Overall inter-rater consistency was high for the total EQIP scores.

**Conclusion:**

Although all the LLMs evaluated were able to generate psychoeducational information about EMDR, substantial differences were observed between the models in respect of informational quality and readability. The findings suggest that psychoeducational quality depends more strongly on the language model used than on the specific EMDR-related topic being addressed. LLMs may serve as supplementary psychoeducational resources but should not be considered as substitutes for professional psychotherapy or individualized clinical care.

## 1 Introduction

Eye Movement Desensitization and Reprocessing (EMDR) is a structured psychotherapeutic approach, the efficacy of which has been established in the treatment of trauma-related psychiatric disorders, particularly post-traumatic stress disorder (PTSD) (1). Despite the high prevalence of traumatic experiences worldwide, socioeconomic barriers and geographical disparities limit access to specialized psychotherapeutic care (2). These challenges have encouraged many individuals to seek mental health information from digital resources, among which artificial intelligence (AI)-based large language models (LLMs) such as ChatGPT, Gemini, and DeepSeek have become increasingly prominent (3, 4). The increasingly widespread use of these systems has led to individuals consulting them not only for general mental health information but also for explanations of psychotherapeutic interventions, treatment expectations, and self-directed psychoeducational guidance.

With the greater public use of these technologies, concerns have emerged regarding the accuracy, completeness, readability, and clinical appropriateness of AI-generated health information (4, 5). The quality of psychoeducational information can influence the attitudes of individuals toward treatment, expectations, and help-seeking behaviors (5). As with other psychotherapies, incomplete, inaccurate, or contextually inadequate information about EMDR, selected as the example therapeutic modality in this study, may contribute to misconceptions about the nature and application of the therapy. The evaluation of AI systems requires consideration of not only the accuracy of the information provided but also whether that information is comprehensible to users with varying levels of education, health literacy, and emotional state (5).

Recent literature has demonstrated a growing interest in evaluating the performance of LLMs across medical and psychiatric topics, with emerging evidence suggesting that AI-generated psychoeducational content may vary considerably in quality and readability (5–7). However, most of the research has focused primarily on psychiatric disorders or diagnostic processes, with comparatively less attention paid to the quality of AI-generated content related to specific psychotherapeutic interventions (4, 8). Unlike generic health information, psychoeducation regarding trauma-focused modalities such as EMDR requires careful clinical contextualization to avoid misunderstandings regarding stabilization strategies or therapeutic expectations.

To address this gap in the literature, EMDR was selected as a representative and highly structured psychotherapeutic approach to evaluate the capabilities of current AI systems. The aim of this study was to evaluate the informational quality and readability of the responses generated by three widely used large language models (ChatGPT, Gemini, and DeepSeek) to publicly searched questions regarding EMDR therapy. The quality, accessibility, and consistency of the AI-generated psychoeducational content were compared using the EQIP instrument and established readability indices. Based on the statistical design and structured nature of the evaluated modality, the following specific hypotheses were tested:

Hypothesis 1: Informational quality and readability metrics would differ significantly across the evaluated large language models (ChatGPT, Gemini, and DeepSeek).

Hypothesis 2: Informational quality was expected to differ across the four EMDR-related thematic question categories.

Hypothesis 3: Higher informational quality would be significantly associated with longer response length (word count) and greater readability, as reflected by higher Flesch Reading Ease and lower Flesch–Kincaid Grade Level scores.

## 2 Materials and methods

### 2.1 Study design and data collection

This study was designed as a cross-sectional content analysis to evaluate the informational quality and readability of responses generated by three widely used large language models (LLMs), ChatGPT, DeepSeek, and Gemini, to user queries related to Eye Movement Desensitization and Reprocessing (EMDR) therapy. Data were collected between December 15 and December 20, 2025. No human participants or animals were involved in the study, and all analyses were performed exclusively on responses generated by publicly accessible AI systems.

ChatGPT (powered by the GPT-4o engine), Gemini (powered by the Gemini 1.5 Pro model), and DeepSeek (powered by the DeepSeek-V3 architecture) were selected for evaluation because they represented the most widely used, advanced, and publicly accessible large language models during the study period. These systems are commonly used by the public to obtain information on a broad range of topics, including health- and mental health–related concerns. To minimize any potential contextual influence from previous interactions, browser histories and session records were cleared prior to data collection and after each session with all prompts entered in newly initiated sessions.

Google Trends was used as the primary search tool to identify EMDR-related search queries. Searches were conducted between December 15 and December 20, 2025, covering the preceding 12-month period, with the geographic setting set to “Worldwide,” the search type set to “Web Search,” and the category restricted to “Health/Mental Health.” The search terms “EMDR,” “EMDR therapy,” and “how does EMDR work” were entered into Google Trends. Both “Top” and “Rising” related queries and related topics were reviewed and extracted for further evaluation. The questions were derived from top and rising EMDR-related search queries identified through Google Trends, with the aim of grounding question selection in public search behavior rather than researcher-generated questions. The first researcher converted the identified search queries into patient-facing question formats and removed duplicate or semantically overlapping items. The second researcher then independently reviewed all candidate questions for content relevance, clarity, and comprehensibility. Any differences of opinion between the researchers were resolved through discussion and consensus. The resulting questions were organized into four thematic categories—EMDR, EMDR and Trauma, EMDR and Anxiety, and EMDR and Depression. After the four thematic categories were established, five questions were intentionally selected from each category through consensus between the two researchers to ensure balanced representation across the categories, resulting in a final set of 20 unique EMDR-related questions for analysis. Google Trends therefore served as a supporting methodological tool for grounding question selection in public search behavior rather than as a primary analytic dataset or an attempt to rank the most frequently searched EMDR questions. Because Google Trends functioned in this study as a question-generation aid rather than as a primary source of quantitative evidence, normalized Trends interest scores were not systematically recorded, and the underlying search export was not archived as supplementary material; instead, the search parameters, terms, and selection workflow reported above are provided in full to enable methodological transparency and reproducibility.

All the AI-generated responses included in the study were collected in a synchronized manner between December 15 and December 20, 2025. The responses were obtained using the most recent model versions and active subscription plans available during the data collection period. ChatGPT responses were generated through the official ChatGPT web interface, Gemini responses through the Google Gemini web interface, and DeepSeek responses through the official DeepSeek web interface. A new chat session was initiated for each question to minimize the potential influence of contextual memory and carryover effects, and to reduce the impact of response variability across sessions and assess the consistency of model outputs, each question was submitted independently in three separate chat sessions for each model. The prompting approach was standardized, where each of the 20 selected questions (e.g., “What is EMDR?”) was submitted directly as a raw query without any system-level persona, pre-conditioning, or role-play instructions, ensuring that the outputs reflected the default public performance of the models. All the questions were submitted in English. No VPN, geographic location masking, or region-specific interface settings were used during the data collection. All the generated responses were archived and subsequently analyzed for informational quality and readability. Each response was treated as an independent observation, resulting in a total dataset of 180 responses (20 questions × 3 models × 3 independent sessions).

### 2.2 Quality assessment

The informational quality of all the AI-generated responses was evaluated using the 20-item short version of the Ensuring Quality Information for Patients (EQIP) instrument (7). EQIP is a validated tool developed to assess the quality, presentation, and adequacy of patient information materials. EQIP was selected because, unlike readability formulas alone, it captures multiple substantive dimensions of written health information—including content, identification, structure, and risk communication—that are directly relevant to psychoeducational material, and because it has previously been applied to evaluate LLM-generated medical content, allowing comparability with earlier studies (19, 20). As EQIP was originally developed and validated for written patient-information leaflets rather than for conversational AI-generated text, some items may not map perfectly onto LLM output—a limitation also explicitly noted by prior EQIP-based evaluations of AI chatbots (19); this potential mismatch is addressed further as a limitation below. Each item is scored as 1 (“yes”), 0.5 (“partly”), or 0 (“no”). Items that could not be meaningfully evaluated because of the nature of the content were coded as “Not Applicable” (NA) and excluded from the score calculations (7).

All the EQIP evaluations were conducted independently by two board-certified psychiatrists, who were blinded to each other’s ratings. For each response, only items not rated as NA were considered applicable items. To account for variation in the number of applicable items across responses, a percentage EQIP score was calculated using the following formula:


EQIP percentage score = (Total EQIP score / Number of applicable items) × 100

Higher scores indicated higher informational quality. Inter-rater reliability was evaluated using weighted Cohen’s kappa coefficients for individual EQIP items. Associations between the total EQIP scores assigned by the two raters were assessed using Spearman’s rank-order correlation and Pearson’s product–moment correlation coefficients.

### 2.3 Readability analysis

The readability of the AI-generated responses was assessed using the Flesch–Kincaid Grade Level (FKGL) and Flesch Reading Ease (FRE) indices (9). The FKGL estimates the educational level required to understand a given text, with lower scores indicating greater readability. The FRE evaluates readability on a scale ranging from 0 to 100, with higher scores reflecting easier-to-read content (9).

The readability analyses were performed using automated natural language processing (NLP)-based assessment methods. The FKGL and FRE scores were calculated using the Python programming language and the textstat library. These indices were selected because they are among the most widely used and validated readability measures in health communication research, enabling comparisons with previous studies that have evaluated patient-oriented health information and AI-generated medical content (5, 10).

### 2.4 Categorization of questions

To facilitate subgroup analyses, all the questions included in the study were classified into four thematic categories according to the primary content focus. The categories were developed based on the thematic characteristics of the questions by two researchers independently and the final forms were agreed through consensus. The distribution of questions across the categories is presented in **Table 1**.

**Table 1:** EMDR-related questions identified through google trends and their thematic categories.

No.	EMDR-related question	Thematic category
1	What is EMDR?	EMDR
2	How is EMDR done?	EMDR
3	How many sessions does EMDR take?	EMDR
4	Who are EMDR therapists?	EMDR
5	Can EMDR be applied to everyone?	EMDR
6	Does EMDR treat trauma?	EMDR and Trauma
7	How does EMDR help with trauma?	EMDR and Trauma
8	Does EMDR work for childhood trauma?	EMDR and Trauma
9	Is EMDR effective for severe trauma?	EMDR and Trauma
10	Is EMDR used for post-traumatic stress disorder (PTSD)?	EMDR and Trauma
11	Does EMDR help with anxiety?	EMDR and Anxiety
12	Does EMDR work for panic attacks?	EMDR and Anxiety
13	Is EMDR effective for social phobia?	EMDR and Anxiety
14	Is EMDR used for generalized anxiety disorder (GAD)?	EMDR and Anxiety
15	Can EMDR be a drug-free treatment for anxiety?	EMDR and Anxiety
16	Does EMDR help with depression?	EMDR and Depression
17	Does EMDR work for trauma-related depression?	EMDR and Depression
18	Can EMDR be used together with medication?	EMDR and Depression
19	Does depression go away after EMDR?	EMDR and Depression
20	Does EMDR change negative thoughts?	EMDR and Depression

EMDR: Questions addressing the definition of EMDR, its theoretical foundations, procedural characteristics, treatment process, and general information regarding its application.

EMDR and Trauma: Questions focusing on the effects of EMDR on traumatic experiences, traumatic memories, and trauma-related emotional or physical responses.

EMDR and Anxiety: Questions examining the potential role of EMDR in the management of anxiety symptoms, anxiety disorders, and anxiety-related triggering experiences.

EMDR and Depression: Questions related to the use of EMDR in addressing depressive symptoms, adverse life experiences, negative self-beliefs, and emotional processes associated with depression.

### 2.5 Statistical analysis

Data obtained in the study were analyzed statistically using IBM SPSS Statistics vn.27 and R statistical software. Descriptive statistics were calculated as mean ± standard deviation values (SD) for EQIP scores, word count, Flesch-Kincaid Grade Level, and Flesch Reading Ease scores. Prior to the primary statistical analysis, readability metrics were computed for each AI-generated response, and these were included in the dataset.

To examine differences in informational quality, a linear mixed-effects model was conducted in the R program. The total EQIP score was entered as the dependent variable, the AI model and question category were included as fixed effects, and the question ID was specified as a random intercept. *Post hoc* pairwise comparisons with Bonferroni correction were performed and Cohen’s d values were reported as effect size estimates.

Pearson correlation analysis was used to examine the relationships between informational quality, response length, and readability metrics. For model-level descriptive comparisons, one-way analysis of variance (ANOVA) was used to compare the AI models in terms of EQIP scores, word count, Flesch-Kincaid Grade Level, and Flesch Reading Ease scores. The linear mixed-effects model was considered the primary inferential analysis for informational quality, accounting for the clustering of responses by question ID. Inter-rater reliability was assessed using Pearson correlation, Spearman’s rank correlation, and weighted kappa coefficients. A value of p<0.05 was accepted as the level of statistical significance in all the analyses.

## 3 Results

### 3.1 Quality assessment

A total of 180 responses generated by three large language models (ChatGPT, DeepSeek, and Gemini) across 20 EMDR-related questions were included in the final analysis. The responses were evaluated in terms of informational quality using the Ensuring Quality Information for Patients (EQIP) instrument, readability using the Flesch–Kincaid Grade Level (FKGL) and Flesch Reading Ease (FRE) indices, and response length was measured by word count.

The overall analysis of 180 responses yielded a mean EQIP score of 56.08± 4.43, reflecting the relative informational quality of the responses across the evaluated models. Of the three models evaluated, Gemini achieved the highest mean EQIP score (60.06± 2.63), followed by DeepSeek (57.01 ± 2.48) and ChatGPT (51.18 ± 2.27). In respect of readability, DeepSeek exhibited the lowest mean FKGL score (12.83 ± 1.36) and the highest mean FRE score (34.97 ± 7.39). DeepSeek generated the longest responses on average (229.63 ± 27.30 words). ChatGPT produced the shortest responses (205.00 ± 8.12 words) and the highest grade-level requirements overall (**Table 2**).

**Table 2:** Descriptive statistics of EQIP and readability metrics according to the AI models.

Model	EQIP score M (SD)	Word count M (SD)	FKGL M (SD)	FRE M (SD)
ChatGPT	51.18 (2.27)	205.00 (8.12)	14.29 (1.00)	27.28 (5.92)
DeepSeek	57.01 (2.48)	229.63 (27.30)	12.83 (1.36)	34.97 (7.39)
Gemini	60.06 (2.63)	214.60 (12.59)	13.33 (1.56)	28.32 (7.59)
Total	56.08 (4.43)	216.41 (20.57)	13.48 (1.45)	30.19 (7.76)

FKGL, Flesch-Kincaid Grade Level; FRE, Flesch Reading Ease; M, Mean; SD, Standard Deviation.

A linear mixed-effects model (LMM) was formed to examine the predictors of psychoeducational information quality (**Table 3**). It was seen that the AI model significantly predicted informational quality. Compared with ChatGPT, both Gemini (β = 8.88, p<0.001) and DeepSeek (*β* = 5.83, p< 0.001) achieved significantly higher EQIP scores, with Gemini demonstrating the highest overall quality performance. Question category was not found to be significantly associated with EQIP scores, as no significant differences were observed for EMDR and Depression (*β* = 1.41, p= 0.107), EMDR and Anxiety (*β* = 0.53, p= 0.542), or EMDR and Trauma (*β* = 0.50, p= 0.565) relative to general EMDR questions.

**Table 3:** Linear mixed-effects model predicting total EQIP scores.

Fixed effects	Estimate (β)	SE	95% CI	z	*P*
Intercept (ChatGPT, EMDR General)	50.57	0.66	[49.27, 51.86]	76.62	< 0.001*
Model (Ref = ChatGPT)					
Gemini	8.88	0.40	[8.09, 9.66]	22.23	< 0.001*
DeepSeek	5.83	0.40	[5.05, 6.62]	14.61	< 0.001*
Category (Ref = EMDR General)					
EMDR and Depression	1.41	0.88	[-0.30, 3.13]	1.61	0.107
EMDR and Anxiety	0.53	0.88	[-1.18, 2.25]	0.61	0.542
EMDR and Trauma	0.50	0.88	[-1.21, 2.22]	0.58	0.565
Random Effects	Variance				
Question ID (Intercept)	1.38				
Residual	4.79				

CI, confidence interval; SE, standard error. Log-Likelihood = -404.50. *P <0.05.

The *post hoc* pairwise comparisons revealed significant differences among all the AI models in respect of the quality of psychoeducational information generated. As presented in **Table 4**, Gemini produced significantly higher EQIP scores than ChatGPT (Δ = 8.88, SE = 0.40, 95% CI [8.09, 9.66], p< 0.001, d = 3.61) and DeepSeek (Δ = 3.05, SE = 0.40, 95% CI [2.26, 3.84], p< 0.001, d = 1.19). DeepSeek achieved significantly higher EQIP scores than ChatGPT (Δ = 5.83, SE = 0.40, 95% CI [5.05, 6.62], p< 0.001, d = 2.45). Bonferroni-adjusted pairwise comparisons indicated that all between-model differences remained statistically significant. Effect size estimates ranged from large to very large (Cohen’s d> 1.0).

**Table 4:** Post-Hoc pairwise comparisons of AI models based on the linear mixed model (LMM).

Comparison pair	Estimate (Δ)	SE	95% CI	Adjusted p	Cohen’s d
Gemini vs. ChatGPT	8.88	0.40	[8.09, 9.66]	< 0.001*	3.61
DeepSeek vs. ChatGPT	5.83	0.40	[5.05, 6.62]	< 0.001*	2.45
Gemini vs. DeepSeek	3.05	0.40	[2.26, 3.84]	< 0.001*	1.19

P values were adjusted using the Bonferroni correction method. Cohen’s d values indicate effect size magnitude. *P <0.05.

### 3.2 Categorization of questions

*Post hoc* pairwise comparisons were conducted to examine differences among EMDR-related question categories within the linear mixed-effects model (**Table 5**). Following Bonferroni correction for multiple comparisons, none of the pairwise comparisons reached statistical significance (all adjusted p values = 1.000). Specifically, no significant differences were identified between EMDR and Depression compared to general EMDR questions (Δ = 1.41, SE = 0.88, 95% CI [-0.30, 3.13], d = 0.62), EMDR and Anxiety compared to general EMDR questions (Δ = 0.53, SE = 0.88, 95% CI [-1.18, 2.25], d = 0.24), or EMDR and Trauma compared to general EMDR questions (Δ = 0.50, SE = 0.88, 95% CI [-1.21, 2.22], d = 0.22). No statistically significant differences were determined among the remaining subcategories. Although several comparisons yielded small to moderate effect sizes, none remained statistically significant after adjustment for multiple testing.

**Table 5:** Post-Hoc pairwise comparisons of question categories based on the linear mixed model (LMM).

Comparison pair	Estimate (Δ)	SE	95% CI	Adjusted p	Cohen’s d
EMDR and Depression vs. EMDR General	1.41	0.88	[-0.30, 3.13]	1.000	0.62
EMDR and Depression vs. EMDR and Anxiety	0.88	0.88	[-0.83, 2.59]	1.000	0.38
EMDR and Trauma vs. EMDR General	0.50	0.88	[-1.21, 2.22]	1.000	0.22
EMDR and Trauma vs. EMDR and Anxiety	-0.03	0.88	[-1.74, 1.68]	1.000	-0.01
EMDR and Anxiety vs. EMDR General	0.53	0.88	[-1.18, 2.25]	1.000	0.24
EMDR and Depression vs. EMDR and Trauma	0.91	0.88	[-0.80, 2.62]	1.000	0.40

CI, confidence interval. P values were adjusted for 10 multiple comparisons using the Bonferroni correction method. *P <0.05.

### 3.3 Readability analysis

Correlation analyses revealed several statistically significant associations among informational quality, readability metrics, and response length (**Table 6**). The EQIP scores were found to be positively correlated with word count (r = 0.243, 95% CI [0.101, 0.375], p = 0.001). The EQIP scores demonstrated a moderate negative correlation with Flesch–Kincaid Grade Level (FKGL; r = −0.338, 95% CI [−0.463, −0.201], p< 0.001) and a positive correlation with Flesch Reading Ease (FRE; r = 0.150, 95% CI [0.004, 0.291], p = 0.045). FKGL and FRE exhibited a very strong negative correlation (r = −0.901, 95% CI [−0.924, −0.871], p< 0.001). Word count was negatively correlated with FKGL (r = −0.232, 95% CI [−0.365, −0.089], p = 0.002) and positively correlated with FRE (r = 0.278, 95% CI [0.138, 0.407], p<0.001).

**Table 6:** Correlations between information quality and readability metrics.

Relationship	r	95% CI	*P*
EQIP Score vs. Word Count	0.243	[.101,.375]	0.001*
EQIP Score vs. FKGL	-0.338	[-.463, -.201]	< 0.001*
EQIP Score vs. FRE	0.150	[.004,.291]	0.045*
FKGL vs. FRE	-0.901	[-.924, -.871]	< 0.001*
Word Count vs. FKGL	-0.232	[-.365, -.089]	0.002*
Word Count vs. FRE	0.278	[.138,.407]	< 0.001*

N = 180. Confidence intervals were computed using Fisher’s z-transformation. *P <0.05.

For model-level descriptive comparisons, one-way ANOVA showed statistically significant differences among the AI models across all evaluated metrics, including informational quality, response length, and readability measures (see **Table 7**). Significant between-group differences were observed for informational quality (EQIP), F(2, 177) = 200.49, p< 0.001, response length (word count), F(2, 177) = 28.62, p< 0.001, Flesch–Kincaid Grade Level (FKGL), F(2, 177) = 18.57, p< 0.001, and Flesch Reading Ease (FRE), F(2, 177) = 21.29, p< 0.001.

**Table 7:** Linguistic and readability metrics variance across AI models.

Metric	F value	df	*P* value
Informational Quality (EQIP)	200.49	(2, 177)	< 0.001*
Response Length (Word Count)	28.62	(2, 177)	< 0.001*
Flesch-Kincaid Grade Level (FKGL)	18.57	(2, 177)	< 0.001*
Flesch Reading Ease (FRE)	21.29	(2, 177)	< 0.001*

df, degrees of freedom. Significant differences were found across all metrics using one-way ANOVA tests. *P < 0.05.

DeepSeek generated the longest responses (229.63± 27.30 words) and had the lowest mean FKGL score (12.83 ± 1.36) and the highest mean FRE score (34.97 ± 7.39). The highest mean EQIP score was obtained by Gemini (60.06 ± 2.63), and the lowest by ChatGPT (51.18 ± 2.27). Overall, the descriptive findings indicated notable differences among the AI models in respect of informational quality, response length, and readability characteristics.

The inter-rater analysis showed strong positive associations between the two independent evaluators’ total EQIP scores across all 180 responses (**Table 8**). Spearman’s rank-order correlation revealed a strong positive association between the raters (ρ = 0.852, p < 0.001), indicating substantial consistency in the relative ranking of EQIP scores. Similarly, Pearson’s product–moment correlation showed a strong positive relationship (r = 0.828, p < 0.001), supporting a high degree of consistency between evaluators. These findings indicate a high degree of consistency in the relative and linear patterns of EQIP scoring across the dataset.

**Table 8:** Inter-rater agreement metrics for total EQIP scores.

Metric	Value	*P* value
Spearman’s Rank Correlation *(ρ)*	0.852	< 0.001*
Pearson Correlation *(r)*	0.828	< 0.001*

Inter-rater rank-order and linear consistency were consistently high across the complete dataset (N = 180). *P < 0.05.

Item-level inter-rater reliability analyses demonstrated generally high agreement across most EQIP criteria (**Table 9**). Almost perfect agreement was observed for several psychoeducational information criteria, including addressing special populations (Q18, κ = 0.978), mentioning complementary medicine (Q20, κ = 0.931), and explaining the benefits of the intervention (Q04, κ =0.883). Substantial agreement was identified for providing realistic expectations (Q17, κ = 0.705), whereas defining the target audience (Q02, κ = 0.588) demonstrated moderate agreement.

**Table 9:** Item-level inter-rater agreement (weighted kappa).

EQIP item	Kappa (κ)	Agreement level
Q18 (Addresses special populations)	0.978	Almost Perfect
Q20 (Mentions complementary medicine)	0.931	Almost Perfect
Q04 (Explains benefits of intervention)	0.883	Almost Perfect
Q17 (Provides realistic expectations)	0.705	Substantial
Q02 (Defines target audience)	0.588	Moderate
Q14 (Identifiable author/organization)	0.192	Slight
Q19 (Addresses financial costs)	0.110	Slight
Q15 (Balanced/unbiased information)	0.000	No Agreement
Remaining 12 Items	Uniform (1.000)	Perfect (Zero Variance)

Interpretation of weighted kappa coefficients was based on conventional agreement benchmarks (Landis & Koch). Items with zero variance indicate that both evaluators assigned identically uniform scores across all responses, precluding standard kappa variance estimation but reflecting 100% absolute agreement. *P <0.05.

Lower levels of agreement were observed for identifiable author or organization information (Q14, κ = 0.192) and financial cost information (Q19, κ = 0.110), both indicating slight agreement. The item assessing balanced and unbiased information (Q15, κ = 0.000) showed no agreement between the raters. The remaining 12 EQIP items demonstrated uniform agreement (κ = 1.000) due to the absence of response variability, indicating that both evaluators assigned identical scores across all responses. Although standard kappa variance estimation could not be computed for these items, complete agreement was observed between the raters.

## 4 Discussion

### 4.1 Principal findings

Evaluations were made of the quality and readability of psychoeducational information generated by three large language models (ChatGPT, DeepSeek, and Gemini) in response to publicly searched questions about Eye Movement Desensitization and Reprocessing (EMDR) therapy. Several important findings emerged. First, significant differences were observed in informational quality and readability across the AI models, with Gemini achieving the highest EQIP scores, followed by DeepSeek and ChatGPT, while DeepSeek produced the most readable responses. These findings supported Hypothesis 1, which anticipated differences in informational quality and readability metrics across the evaluated models. Second, no significant differences were observed in informational quality across the four thematic question categories. This finding did not support Hypothesis 2, which anticipated differences in informational quality across the four thematic question categories. Third, higher-quality responses were associated with greater readability and longer response length. Finally, inter-rater analyses demonstrated a high level of consistency in EQIP scoring. Collectively, these findings suggest that the language model generating the response drives differences in psychoeducational information quality more than the specific EMDR-related topic being addressed.

The findings of this study should be interpreted within the broader context of self-directed mental health education. For decades, researchers have explored whether individuals can benefit from bibliotherapy, self-help interventions, and internet-based psychoeducational resources as adjuncts to traditional mental health care (11, 12). The emergence of large language models has introduced a new dimension to this discussion (13, 14). Unlike static educational materials, LLMs provide interactive, individualized, and continuously accessible responses, allowing users to obtain information about mental health conditions and psychotherapeutic interventions in a conversational format (13, 14). Consequently, AI systems are being increasingly used by the public not only as information retrieval tools but also as informal sources of psychoeducation and mental health guidance (13–15). This growing role of AI in psychotherapy-related contexts has led to increasing discussion regarding the opportunities, limitations, and ethical implications of intelligent therapeutic systems in mental health care (13, 15).

EMDR was selected as the focus of this study because it represents a relatively structured and protocol-driven psychotherapeutic approach. Unlike many psychotherapy models that rely heavily on individualized formulations and flexible therapeutic techniques, EMDR features clearly defined procedural stages, standardized treatment principles, and specific therapeutic procedures (1). Therefore, EMDR provides a useful framework for the examination of whether AI-generated psychoeducational information can accurately communicate the essential components of a psychotherapy intervention. The absence of significant differences in information quality across the EMDR-related subtopics suggests that the quality of AI-generated psychoeducational information remained relatively consistent across questions addressing trauma, anxiety, depression, and general aspects of EMDR. However, this finding may also indicate a tendency for LLMs to present psychotherapy-related information at a relatively general level, potentially overlooking clinically important distinctions between different therapeutic applications and patient populations (13). For example, the clinical application of EMDR in trauma-related disorders may differ substantially from its use in depression, anxiety disorders, or more complex clinical presentations requiring protocol modifications and individualized treatment planning (1). While providing general information about a psychotherapeutic approach may be sufficient for introductory psychoeducation, effective psychotherapy often requires attention to clinically nuanced distinctions related to symptom presentation, patient characteristics, treatment goals, and contextual factors. Therefore, the absence of topic-specific variation observed in this study may suggest that current LLMs are more proficient at communicating general psychotherapy concepts than at conveying the complexity of individualized therapeutic applications. From a clinical perspective, this distinction is important because the ability to describe a psychotherapeutic approach does not necessarily translate into the ability to provide individualized guidance for appropriate application in real-world clinical contexts.

The clinical implications of these findings warrant careful consideration. The interactive format of AI systems may improve access to psychoeducational information and support mental health literacy (14, 16). The findings of this study suggest that LLMs have potential as supplementary psychoeducational tools for individuals seeking information about psychotherapeutic interventions. However, important differences were observed in informational quality across the models, and the generated content often remained at a relatively general level. These findings indicate that the quality and depth of psychotherapy-related information are not consistent across platforms. Consequently, clinicians and users should exercise caution when relying on AI systems as sources of psychotherapy-related guidance (13, 14). Taken together, the results suggest that current LLMs can provide psychotherapy-related psychoeducational information, but the informational quality of their responses depends on both the model used and the complexity of the information being communicated.

The broader question of whether AI systems can meaningfully support psychotherapeutic processes extends beyond the provision of information alone (13, 14). Effective psychotherapy requires multiple competencies, including empathic engagement, individualized case formulation, clinical judgment, risk assessment, and responsiveness to complex emotional experiences (1, 13). Nevertheless, the ability to provide high-quality and understandable psychoeducational information represents an important prerequisite for any system intended to support psychotherapeutic care (14). This issue is particularly important because individuals seeking psychotherapy-related information may differ considerably in their educational background, health literacy, cognitive functioning, and emotional state (14, 16). Consequently, both the accuracy and readability of information are essential for effective communication (16). Although the full range of competencies required for psychotherapy were not evaluated in this study, one important prerequisite was examined with the assessment of the quality and readability of the AI-generated psychoeducational information.

Despite the potential value of LLMs as psychoeducational resources, psychotherapy involves considerably more than providing information (13, 14). Although EMDR is a structured and protocol-based intervention, its effective clinical use requires patient assessment, preparation, individualized case formulation, and continuous clinical judgment (1). Therefore, even information that is accurate and easy to understand may still be insufficient to support treatment-related decisions or the application of psychotherapeutic techniques without professional guidance (13). Generalized AI-generated responses often fail to adequately reflect highly individualized, important factors such as patient readiness, stabilization needs, treatment indications, and protocol adaptations (1, 13).

### 4.2 Comparisons with previous research

The differences observed between the AI models highlight the need for caution when using LLMs as sources of psychotherapy-related psychoeducation (5, 7). Gemini achieved the highest informational quality scores, whereas DeepSeek produced the most readable responses and ChatGPT consistently obtained lower EQIP scores. These findings indicate that the quality of psychotherapy-related information may vary substantially depending on the model used. Similar differences between LLMs have been reported in previous studies evaluating medical and mental health information (5, 7). It is worth noting that the present study evaluated *de novo* LLM-generated psychoeducational content rather than LLM-assisted simplification of existing materials. In contrast, prior work has explicitly prompted LLMs to rewrite or simplify pre-existing patient-education texts, and such approaches have reported measurable readability gains (21). This suggests that prompt design and task framing, in addition to the underlying model itself, are important determinants of output quality. The current study findings are also consistent with a previous study by the current authors on bipolar disorder psychoeducation, which demonstrated considerable variability in information quality across language models (6). Taken together, the available evidence suggests that individuals seeking psychotherapy-related information from AI systems may receive markedly different levels of informational quality depending on the platform consulted (5–7). The reasons for the superior informational quality provided by Gemini in this study remain unclear, and whether this advantage is consistent across different domains or specific to psychotherapy-related content also remains unknown. Further studies are needed to determine whether differences in training data, model architecture, alignment procedures, or response-generation strategies contribute to the observed variability across models.

An additional noteworthy finding was the positive relationship between informational quality and readability. Responses with higher EQIP scores tended to be longer, easier to read, and associated with lower grade-level requirements. This pattern suggests that higher-quality AI-generated content does not necessarily require greater linguistic complexity, but that authors or systems can keep information both comprehensive and accessible when they present it in a clear and structured manner (14, 16). From a psychoeducational perspective, this finding is particularly important because both readability and informational quality contribute to effective patient education (16). This observation may also help to explain the performance of DeepSeek, which achieved the highest readability scores despite generating the longest responses. Previous studies have similarly suggested that structured, user-oriented information may be easier to understand than condensed technical explanations (16, 17). Although response structure was not directly evaluated in this study, differences in how models organized information may have contributed to the observed variation in readability across platforms.

Despite the relatively favorable readability performance of DeepSeek, all the evaluated models generated responses that exceeded the commonly recommended readability thresholds for patient education materials. International health communication guidelines generally recommend that authors write patient-facing information at approximately the sixth to eighth grade reading level (15, 16), whereas the responses evaluated in this study corresponded to late high-school or university-level reading requirements. It should be acknowledged that EMDR, as a technically structured and mechanism-based psychotherapeutic modality, may inherently require a somewhat higher reading level than general health information to be conveyed accurately. Some degree of elevated complexity may therefore be difficult to avoid entirely without loss of clinical precision. Future studies should directly examine whether simplified language can be used to describe EMDR and similarly structured interventions without compromising informational accuracy. Some technical procedures within EMDR may be particularly difficult to describe in very simple terms without losing important clinical detail. Such research could, for example, compare simplified and standard AI-generated explanations of EMDR and check whether patients understand them equally well and equally accurately. This finding is particularly important in mental health settings, where psychological distress, reduced concentration, cognitive burden, or emotional dysregulation may limit an individual’s ability to process complex information (14). Consequently, readability represents more than a communication issue; it directly impacts patient understanding and informed help-seeking (15, 16). In the context of EMDR, texts often introduce individuals to concepts such as bilateral stimulation, memory reprocessing, stabilization procedures, and trauma-related mechanisms (1). Without sufficient clinical context, users may misunderstand these concepts, which can then potentially lead to inaccurate expectations about the therapeutic process and treatment goals (1, 13). Therefore, even when models generate factually accurate information, the practical value is limited if it is not presented in a manner that is accessible to the intended audience (15, 16).

These concerns may be particularly relevant in the context of psychotherapy (13). Unlike many forms of general health information, psychotherapy-related content may influence not only knowledge but also treatment expectations and help-seeking behavior (11, 12, 14). In the case of EMDR, misunderstandings regarding preparation procedures, treatment indications, or expected therapeutic outcomes may lead to inaccurate assumptions about the therapeutic process or unrealistic expectations regarding treatment (1). Although behavioral outcomes were not evaluated in this study, the observed differences in informational quality across models suggest that caution is warranted when AI-generated content is used as a source of psychotherapy-related psychoeducation (6, 8).

The reliability analyses provide additional support for the consistency of the present findings. The independent evaluators demonstrated strong consistency in overall EQIP scoring, indicating that they applied the assessment criteria consistently across the dataset. The evaluators showed lower levels of agreement for a small number of items related to balance, neutrality, and contextual presentation. These aspects of AI-generated responses may be more difficult to evaluate consistently because they involve a greater degree of subjective judgment than factual content. Previous studies evaluating AI-generated health information have similarly reported variability in the quality, readability, and appropriateness of AI-generated responses (18). Overall, the consistency observed between the evaluators supports the reliability of the quality assessments reported in this study.

### 4.3 Limitations

This study had several limitations to be considered when interpreting the findings. First, the study evaluated only three large language models and therefore may not reflect the performance of other AI systems currently available. Second, AI models are continuously updated, and the quality of generated responses may change over time. Consequently, the present findings should be interpreted as a snapshot of model performance during the study period. Third, the study assessed informational quality and readability rather than actual user comprehension, clinical decision-making, or behavioral outcomes. In addition, the study did not compare AI-generated responses with psychoeducational materials developed by mental health professionals, so it remains unclear whether AI-generated psychoeducational information is comparable in quality to educational materials developed by mental health professionals. Therefore, the EQIP scores reported in this study should be interpreted as reflecting the relative performance of the AI models compared with one another, rather than as an absolute indicator of adequate or inadequate informational quality. Additionally, although EQIP has previously been applied to AI-generated content, it was originally developed and validated for static, professionally authored patient-information leaflets. Twelve of the twenty EQIP items showed uniform scoring across raters (κ = 1.000), which may reflect a structural mismatch between certain EQIP items and the nature of conversational AI-generated text rather than a genuine absence of variation in informational quality, and this structural mismatch should be taken into account when interpreting the findings of this study. Future studies may therefore benefit from adapting the EQIP framework specifically for AI-generated conversational health information, with revised or additional items that better capture the characteristics of dynamically generated content. Such research should also examine how patients interpret, understand, and apply psychotherapy-related information obtained from AI systems. Furthermore, because the final set of questions and their thematic categorization were determined by the researchers—albeit through a structured, Google Trends–based process involving independent review and consensus—the possibility of selection bias in the specific composition of the question set cannot be entirely excluded, and the resulting set does not cover the entirety of possible EMDR-related information-seeking patterns. However, as the primary aim of the study was to compare model performance, using realistic, publicly searched queries rather than to comprehensively catalog all such patterns, the exact composition of the question set is likely to have limited influence on the overall pattern of between-model differences reported here. As only English-language questions and responses were evaluated, the informational quality and readability findings cannot be generalized to outputs generated in other languages or cultural contexts. Future studies should examine AI-generated psychoeducational responses across different languages and cultural settings, as both the informational quality and readability of LLM outputs may vary depending on the linguistic and cultural characteristics of the training data and user queries. Moreover, while Google Trends provided a systematic approach to identifying public interest, it lacks sociodemographic and clinical granularity, meaning the extracted queries may not fully reflect the specific psychoeducational needs of diverse patient populations. Finally, the present investigation focused specifically on EMDR, a relatively structured psychotherapy approach. Although these findings may theoretically be considered valid for other therapeutic modalities as well, whether this is the case remains uncertain. There remains need for future studies to examine AI-generated psychoeducational information across a broader range of psychotherapies, including less structured and more interpretive approaches such as psychodynamic and psychoanalytic therapies.

## 5 Conclusion

In conclusion, the results of this study, which examined EMDR therapy, demonstrated that large language models can generate psychoeducational information about psychotherapy, but the quality and readability of this information varied considerably across the models. Although some systems produced relatively high-quality responses, overall readability levels remained above the commonly recommended standards for patient education materials. These findings suggest that current LLMs may be useful as supplementary sources of psychotherapy-related psychoeducation but should not be viewed as substitutes for professional psychotherapy, clinical judgment, or individualized mental health care. More broadly, the results highlight both the promise and the current limitations of AI systems in mental health communication. As large language models become increasingly integrated into routine mental health information seeking, improving the quality and accessibility of AI-generated psychotherapy-related psychoeducational information should remain an important priority for researchers, clinicians, and developers.

## Data Availability

The raw data supporting the conclusions of this article will be made available by the authors, without undue reservation.
